# Clinical characteristics of adverse reactions to nonionic low osmolality contrast media in patients transferred from the CT room to the emergency room

**DOI:** 10.1186/s40064-016-2380-5

**Published:** 2016-06-30

**Authors:** Sang Ook Ha, Dae Yong Kim, You Dong Sohn

**Affiliations:** Department of Emergency Medicine, Hallym University Sacred Heart Hospital, Hallym University Medical Center, 22, Gwanpyeong-ro 170 Beon-gil, Dongan-gu, Anyang-si, Gyeonggi-do 431-070 Korea; Department of Emergency Medicine, University of Eulji College of Medicine and Eulji Medical Center, Seoul, Korea

**Keywords:** Adverse reaction, Contrast media, Severity

## Abstract

Nonionic low osmolality contrast media (LOCMs) are used universally in computed tomography (CT) imaging. Although adverse reactions due to nonionic LOCMs are a common cause of emergency room (ER) admissions, few studies have investigated these adverse reactions. In the present study, we evaluated the characteristics of patients who were transferred from the CT room to the ER due to adverse reactions to contrast media, and we determined the risk factors for severe adverse reactions. A single-center retrospective study was conducted over a 41-month period. Baseline and clinical characteristics were evaluated and analyzed according to moderate and severe severity. In particular, risk factors of severe reactions were determined using logistic regression analysis. In total, 70 patients were admitted to the ER with adverse reactions due to nonionic LOCMs. Of these, 33 developed a moderate reaction, and 37 developed a severe reaction. Compared with the moderate reaction group, the severe reaction group was older, had higher blood pressures, showed more symptoms indicating the cardiovascular and central nervous system, and developed faster reactions to LOCMs. According to the multivariate logistic regression analysis, the age of the patient and time to onset of reaction demonstrated a statistical relationship with severe adverse reactions. In the receiver operating characteristic analysis, the optimal cutoff values for age and time to onset were 60 years and 5 min. In conclusion, clinicians should be attentive to anaphylaxis due to nonionic LOCM, in particular, for elderly patients aged older than 60 years and a time to reaction onset of less than 5 min.

## Background

Contrast media are currently used for imaging at least one to 70 million times per year in the United States (Christiansen 2005). Computed tomography (CT) examination with administration of contrast media causes a risk of developing adverse reactions to the contrast media. Compared with the use of ionic contrast media, nonionic low osmolality contrast media (LOCMs) have been associated with a reduced overall prevalence of adverse reactions (Grant and Camamo 1997; Jacobs et al. 1998). However, adverse reactions due to nonionic LOCMs remain an important cause of admissions to the emergency room (ER). Furthermore, the lower incidence of adverse reactions resulted in fewer opportunities to develop and maintain these necessary skills (Segal and Bush 2011). In the present study, we evaluated the characteristics of patients transferred from the CT room to the ER with moderate or severe adverse reactions due to nonionic LOCMs and evaluated the risk factors for severe adverse reactions.

## Methods

### Study design and patient selection

This study was approved by the author’s Institutional Review Board for the evaluation and analysis of patient data. Due to the purely observational, retrospective, and non-interventional nature of this study, informed consent was deemed unnecessary and was not obtained. Patient records and information were anonymized and de-identified prior to analysis. Our protocol for adverse reactions to contrast media was as follows: (1) patients with moderate or severe reactions to contrast media received an injection [intravenous antihistamine (4 mg) and methylprednisolone (0.5 mg/kg) with or without intramuscular epinephrine (0.3 mg)] and were transferred from the CT room to the ER. (2) Patients with mild reactions were discharged (with or without antihistamine administration) from the CT room. From January 2010 to May 2013, patients with moderate or severe reactions to contrast media who were transferred from the CT room to the ER were enrolled in the present study. For the inclusion of patients with delayed moderate or severe adverse reactions, the diagnostic terms ‘anaphylaxis’, ‘anaphylactic shock’, and ‘anaphylactic reaction’ provided by the “International Statistical Classification of Diseases and Health-related Problems, 10th revision (ICD 10)” were used to identify the patients. Among various causes, only contrast media were included in the present study. Patients aged younger than 18 years were excluded from the analysis.

### Data collection

Baseline characteristics, including gender; age; vital signs; history of allergy to food, drugs, or contrast agents; morbidity; history of medication; and clinical characteristics, including type of contrast medium, premedication, treatments, symptoms and disposition, were collected as data in the electronic medical records. The records for time to onset of adverse reaction, immediate symptoms and vital signs were reviewed in report form written by the radiology department for moderate or severe adverse reactions according to the hospital policy.

### Comparison of moderate and severe adverse reactions

Patients were grouped into moderate and severe groups according to the severity of the adverse reactions they developed in response to contrast media. The baseline and clinical characteristics of the patients were compared by group. Risk factors for severe reactions were determined using univariate and subsequent multivariate logistic regression analyses. A receiver operating characteristic (ROC) analysis was performed to determine the optimal cutoff values for each potential risk factor.

### Definition and classification of adverse and immediate reactions to contrast media

In the recently published “2013 ACR Manual on Contrast Media—Version 9”, adverse reactions are categorized as allergic-like reactions and physiologic reactions (ACR Committee on Drugs and Contrast Media 2013). However, when the reaction was severe, there were no significant differences in clinical manifestations and treatment methods between the two reaction categories. Therefore, the severity of the adverse reactions for this particular study was classified according to the “ACR Manual on Contrast Media—Version 8”, as shown in Table 1 (ACR Committee on Drugs and Contrast Media 2012). A reaction was identified as immediate if it occurred within the first hour of administration of LOCM and as delayed if it occurred after the first hour had passed.

**Table 1:** Classification of adverse reactions in response to contrast media

Severity	Categories of reactions		
Mild	Nausea, vomiting Cough Warmth Headache Dizziness Tremor	Altered taste Itchiness Pallor Flushing Chills	Perspiration Rash, hives Nasal stuffiness Swelling of the eyes and face Anxiety
Moderate	Tachycardia Bradycardia Hypertension Generalized or diffuse erythema	Dyspnea Bronchospasm Wheezing	Laryngeal edema Mild hypotension
Severe	Laryngeal edema (severe or rapidly progressing) Unresponsiveness	Cardiopulmonary arrest Convulsions	Profound hypotension Clinically manifested arrhythmia

### Selection of contrast media and premedication protocol

Throughout the study, all contrast media used in our center were nonionic LOCMs. The dose, rate, and type of contrast media were determined according to the protocol provided by our center. Contrast media (100–150 mL) were administered at a rate of 2.5–4 mL/s. If a patient had previously developed a moderate or severe reaction to a certain contrast medium, other contrast agents were recommended for the next administration, and premedication [intravenous Peniramin (4 mg at 1 h) and Cortisol (200 mg at 4 and 1 h) prior to contrast administration] were used.

### Statistical analysis

The normality of the data distribution was evaluated using the Kolmogorov–Smirnov test, and the statistical methods used for analysis were selected accordingly. Median values (with 25–75th percentile values in parentheses) were used to present continuous data, which were not normally distributed. Numerical values (with percentile values in parentheses) were used to show categorical data. Categorical data were analyzed using the Chi squared or Fisher exact tests, whereas continuous data were analyzed using the Mann–Whitney U test for two-group unpaired comparisons. Risk factors for severe reactions were determined using multivariate logistic regression analysis, which included all variables that provided a *p* value ≤0.2 in the univariate analysis. Subsequently, a ROC analysis was performed to determine the optimal cutoff values for each potential risk factor. For all comparisons, tests were two-tailed, and *p* values <0.05 were regarded as statistically significant. SPSS 18.0.0 software for Windows (SPSS, Inc., Chicago, IL) was used for all statistical analyses.

## Results

### Baseline characteristics

The baseline characteristics of the patients enrolled in the study are summarized in Table 2. Patients in the severe group were older (55 vs. 59, *p* = 0.068) and had lower blood pressures (110/69 vs. 72/41, *p* < 0.001) compared with those in the moderate group. Between the two groups, there were no significant differences in gender; heart rate; respiratory rate; history of allergy to foods, drugs, or contrast media; morbidity due to hypertension, diabetes mellitus, or asthma; or history of medication. The most widely administered LOCM was Ultravist (34 times); Iopamiro and Xenetix were used infrequently (2 times).

**Table 2:** Baseline characteristics of the enrolled patients

Variables	Moderate (n = 33)	Severe (n = 37)	*p*
Sex (M:F ratio)	17:16	18:19	0.811
Age (year)	55 (45–61)	59 (51–72)	0.068
Initial vital signs			
SBP (mmHg)	110 (104–120)	69 (60–80)	<0.001
DBP (mmHg)	72 (70–80)	41 (35–50)	<0.001
Heart rate (beats/min)	86 (68–96)	75 (71–89)	0.708
Respiratory rate (breaths/min)	20 (18–23)	16 (14–18)	0.400
Previous allergy	15 (45.5)	16 (43.2)	0.853
Drug			0.600
Pyrin	1	1	
Hydrocortisone	0	2	
NSAID	1	0	
Food			0.599
Seafood	1	0	
Pupa	1	0	
Mushroom	0	1	
CT contrast media	12 (36.4)	20 (54.1)	0.138
Morbidity			
Hypertension	13 (39.4)	10 (17.0)	0.271
Diabetes mellitus	5 (15.2)	3 (8.1)	0.462
Asthma	0	0	
Concurrent medications			
Calcium channel blocker	8 (24.2)	8 (21.6)	0.794
ACE inhibitor	8 (24.2)	4 (10.8)	0.135
Beta blocker	3 (9.1)	2 (5.4)	0.661
Type of contrast media			0.436
Iomeron	3	2	
Iopamiro	2	0	
Omnipaque	3	1	
Optiray	2	5	
Pamiray	8	7	
Ultravist	14	21	
Xenetix	1	1	
Contrast change	23 (69.7)	20 (54.1)	0.180

*ACE* angiotensin converting enzyme, *CT* computed tomography, *DBP* diastolic blood pressure, *F* female, *M* male, *NSAID* non-steroid anti-inflammatory drug, *SBP* systolic blood pressure

### Time to onset of reaction and symptoms

The time to onset of reaction was significantly different between the moderate and severe groups (7.0 vs. 4.0 s, *p* < 0.001). The incidence of an immediate reaction that occurred within the first hour was significantly higher for the severe group compared with the moderate group (81.8 vs. 100 %, *p* = 0.008).

The most common symptom was skin-related or mucosal (urticarial and angioedema), followed in decreasing order by cardiovascular, respiratory, central nervous and gastrointestinal system-related symptoms. Relative to the moderate reactions, severe reactions more frequently involved cardiovascular and central nervous system symptoms, as shown in Table 3 (27.3 vs. 67.6 %, *p* < 0.001, and 24.2 vs. 97.3 %, *p* = 0.001).

**Table 3:** Time to onset of reaction and symptoms

Variables	Moderate (n = 33)	Severe (n = 37)	*p*
Time to onset (min)	7.0 (4.0–19.5)	4.0 (2.5–5.0)	<0.001
Time to onset			0.008
<1 h (immediate)	27 (81.8)	37 (100.0)	
>1 h (delayed)	6 (18.2)	0	
Symptoms			
Skin/mucosal	26 (78.8)	32 (86.5)	0.394
Urticaria	24	26	
Angioedema	21	16	
Respiratory	19 (57.6)	16 (43.2)	0.231
Dyspnea	12	14	
Neck tightness	6	2	
Stridor, hoarseness	3	1	
Hypoxia	0	1	
Central nervous system	9 (27.3)	25 (67.6)	0.001
Faint/dizziness	7	18	
Loss of consciousness	2	9	
Seizure	0	4	
Headache	0	1	
Cardiovascular	8 (24.2)	36 (97.3)	<0.001
Hypotension	0	36	
Chest discomfort	7	4	
Tachycardia	1	1	
Gastrointestinal	4 (12.1)	10 (27.0)	0.120
Vomiting	3	8	
Abdominal pain	1	4	
Diarrhea	0	0	

### Premedication, treatment, and outcome

More patients in the severe group received premedication (48.6 %) than those in the moderate group (30.3 %). However, among the pretreated patients, there were no significant differences in the number of adverse reactions between the two groups, as shown in Table 4. To treat adverse reactions, an H_1_ blocker (67 cases) was used most frequently, followed by epinephrine (48 cases), an H_2_ blocker (46 cases), hydrocortisone (38 cases), methylprednisolone (32 cases), and a β_2_ agonist nebulizer (4 cases). Excluding epinephrine, there were no significant differences in the number of medications used for treatment between the two groups. Epinephrine was more widely used to treat patients in the severe group compared with the moderate group (100 vs. 33.3 %, *p* < 0.001). More patients with severe reactions were admitted to the hospital than those with moderate reactions (45.9 vs. 12.1 %, *p* = 0.004). There were no mortalities in either group.

**Table 4:** Pretreatment, treatment, and outcome

Variables	Moderate (n = 33)	Severe (n = 37)	*p*
Pretreatment	10 (30.3)	18 (48.6)	0.118
Antihistamine	10	18	
Hydrocortisone	10	18	
Treatment			
H_1_ blocker	33 (100.0)	34 (91.9)	0.242
H_2_ blocker	23 (69.7)	23 (62.2)	0.507
β_2_ agonist nebulizer	2 (6.1)	2 (5.4)	1.000
Steroid	25 (75.8)	32 (86.5)	0.249
Hydrocortisone	16	22	
Methylprednisolone	14	18	
Dexamethasone	0	0	
Epinephrine	11 (33.3)	37 (100.0)	<0.001
Intramuscular	5	28	
Subcutaneous	3	3	
Intravenous	3	6	
Disposition			0.004
Discharge	26 (78.8)	19 (51.4)	
Admission	4 (12.1)	17 (45.9)	
AMA discharge	3 (9.1)	1 (2.7)	
Death	0	0	

*AMA* against medical advice, *β* ^*2*^ beta receptor 2, *H*_*1*_ histamine receptor 1, *H*_*2*_ histamine receptor 2

### Risk factor of severe adverse reactions

Multivariate logistic regression analysis revealed that, after adjusting for previous adverse events to LOCMs, change in contrast, and pretreatment, the age of the patient and time to onset of the reaction were statistically significant factors for the development of severe reactions, as shown in Table 5 [odds ratio (OR) 1.053, *p* = 0.042, and OR 0.805, *p* = 0.020]. The clinical relevance of these risk factors was further confirmed through subsequent ROC analysis. The areas under the ROC curves were 0.627 [95 % confidence interval (CI) 0.497–0.757, *p* = 0.068] for patient age and 0.756 (95 % CI 0.639–0.873, *p* < 0.001) for time to onset of reaction. The optimal cutoff values for age and time to onset of reaction for severe reactions was 60 years (sensitivity of 43.2 % and specificity of 75.8 %) and 5 min (sensitivity of 72.7 % and specificity of 70.3 %), respectively (data not shown).

**Table 5:** Logistic analysis for the prediction of severe adverse events in response to nonionic LOCMs

Variables	Univariate analysis, OR (95 % CI)	*p*	Multivariate analysis, OR (95 % CI)	*p*
Age (years)	1.049 (1.005–1.096)	0.029	1.053 (1.002–1.106)	0.042
Sex (male)	0.892 (0.349–2.280)	0.811		
Time to onset (min)	0.797 (0.666–0.954)	0.013	0.805 (0.671–0.966)	0.020
Previous allergy to LOCMs	2.059 (0.789–5.376)	0.140		
Change of contrast	0.512 (0.191–1.369)	0.182		
Pretreatment	2.179 (0.815–5.825)	0.121		

*LOCM* low osmolality contrast media, *OR* odds ratio

## Discussion

Use of nonionic LOCMs and enforcement of pretreatment have been proposed as methods to minimize adverse reactions in response to contrast media. Nevertheless, attention must be given to adverse reactions resulting from the use of nonionic LOCMs. If a comprehensive guideline regarding prevention and treatment is not available, such reactions can be life threatening or fatal. In the present study, the baseline and clinical characteristics of patients who developed moderate or severe reactions to contrast media and were transferred from the CT room to the ER were evaluated and compared between moderate and severe groups. Compared with patients with a moderate reaction, those with a severe reaction were older, had higher blood pressures, showed more symptoms indicative of cardiovascular and central nervous system complications, and developed faster reactions to LOCMs. In particular, elderly patients older than 60 years and a time to reaction onset of less than 5 min were risk factors of a severe reaction.

### Risk factors of a severe adverse reaction to LOCMs

Until recently, very few clinical studies have investigated the association between patient age and adverse reactions to contrast media. Shehadi et al. and Lieberman et al. demonstrated that adverse reactions occurred most frequently in patients in their 20s and 50s and least frequently in patients at either end of the age spectrum (Shehadi 1975). Furthermore, a recent study of gadobutrol safety in elderly patients demonstrated no greater incidence of adverse reactions in elderly patients (≥65 years) compared with younger adults (Endrikat et al. 2015). By contrast, Cashman et al. suggested that the mortality was age-related and that the cutoff value for age was 65 years (Cashman et al. 1991). Similar results were obtained in the present study. We suggest that the discordant results stem from the different characteristics between LOCMs, high osmolality contrast media of CT tests, and gadobutrol used for magnetic resonance imaging. Older patients presented a higher risk of a severe reaction, and the cutoff value for age was 60 years. The pulse rates of patients in the severe group were generally lower than those of patients in the moderate group (75 vs. 86, respectively), although this difference was not significant. These results suggest that hypotension in old age may be further complicated by failure of the heart to adequately compensate for vasodilatation.

To date, only a few studies have reported a relationship between an adverse reaction and the time to onset of the reaction, and the results have varied. Kunishima et al. reported that the symptoms of overall adverse reactions to contrast media occurred primarily within 5 min of administration of the contrast media (Kunishima et al. 2009). Hartman et al. and Shehadi found that moderate and severe reactions occurred within 20 min of administration (Hartman et al. 1982; Shehadi 1985). In the present study, the time to onset of reaction differed significantly between the moderate and severe groups. Patients with a severe reaction developed symptoms within 15 min of LOCM administration, and those with a moderate reaction developed symptoms within 72 min. In addition, the cutoff value for the time to onset of reaction was 5 min for severe reactions. These findings suggest that it is necessary to observe patients for at least 15 min after they have undergone a CT examination with contrast media. Clinicians should monitor the occurrence of rapid deterioration, particularly if symptoms develop within 5 min of the administration of contrast media.

According to previous studies, risk factors associated with adverse reactions to contrast media are a history of allergy to foods, drugs, or contrast media and concurrent use of a beta-blocker. In the present study, we evaluated whether these risk factors could influence the severity of adverse reactions. In another study, the incidence of adverse reactions in patients with a previous allergy history was 4.5 times higher than that in patients with no allergy history (Davenport et al. 2009). However, the results of the present study demonstrated no statistical relationship between severity of an adverse reaction and previous allergy history to contrast media, foods, or medication.

### Effects of premedication and change in contrast media

Pretreatment is not necessary in patients with no history of adverse reactions to contrast media (Dawson and Sidhu 1993; Radhakrishnan et al. 2005). According to Freed and Davenport, when a pretreated patient develops a breakthrough reaction, the severity of the reaction is similar to that of the adverse reaction previously experienced by the patient (Freed et al. 2001; Davenport et al. 2009). However, the results of the present investigation revealed no significant relationship between premedication and severity of adverse reactions. By contrast, a greater number of patients in the severe group were pretreated compared with the moderate group. We propose that our contrasting results are due to the inadequate dosage and frequency in our protocol compared with the previous protocol. Thomsen et al. recommended the use of different contrast media in further studies investigating patients with a history of reactions to contrast media (Thomsen and Bush 1998). The results of the present study revealed a two-fold decrease in the incidence of severe reactions when the contrast media administered to patients differed from those to which the patients showed a previous allergy, although this change was not statistically significant.

### Treatment of adverse reactions

The ACR manual on contrast media recommends the use of antihistamines, steroids and epinephrine to treat moderate and severe reactions. In the present study, there was no significant difference in the number of patients who were treated with an antihistamine and a steroid in the moderate and severe groups. There was also no significant difference in the dosages of antihistamine between the two groups (1.36 vs. 1.44, *p* = 0.525). Three patients in the severe group had only an epinephrine injection without an antihistamine and steroid, through a mistake of the medical team in the CT room. These patients showed improvement of symptoms after epinephrine administration; hence, the additional antihistamine and steroid were unnecessary in the ER. However, there was a large difference in the number of patients who were treated with epinephrine between the moderate and severe groups (33.3 vs. 100.0 %, relatively). Specifically, the use of epinephrine was more than twice as common in the severe group (6.1 vs. 45.9 %, *p* < 0.001), and continuous intravenous administration of epinephrine was required for four patients in the severe group. The lower use of epinephrine in the moderate group than the severe group may be explained by the tendency of clinicians to hesitate when prescribing epinephrine to patients without significant hypotension and hypoxia. Through proper education, clinicians should be encouraged to increase the use of epinephrine for the treatment of moderate adverse reactions.

The limitations of this study are that it was designed retrospectively, data were collected from report forms and electronic medical records, and selection bias could not be eliminated. Another limitation is that patients who had mild reactions or who were not admitted to the ER were not enrolled in the study, rendering it impossible to evaluate the overall clinical characteristics of adverse reactions to nonionic LOCMs. Finally, a small sample size was assessed because the patient data were collected from a single hospital.

## Conclusion

Compared with patients with a moderate reaction, those with a severe reaction were older, had higher blood pressures, showed more symptoms indicative of cardiovascular and central nervous system complications, and developed faster reactions to LOCMs. In particular, elderly patients older than 60 years and a time to reaction onset of less than 5 min were risk factors of a severe reaction.
